# Addressing knowledge gaps: the key role of community health workers and healthcare providers in human papillomavirus prevention and vaccine uptake in a border community

**DOI:** 10.3389/fpubh.2023.1243539

**Published:** 2023-09-27

**Authors:** Eva M. Moya, Araceli Garcia, Amy Joyce Ponder, Gabriel Frietze

**Affiliations:** ^1^Department of Social Work, College of Health Sciences, The University of Texas at El Paso, El Paso, TX, United States; ^2^Border Biomedical Research Center, The University of Texas at El Paso, El Paso, TX, United States; ^3^School of Pharmacy, College of Health Sciences, The University of Texas at El Paso, El Paso, TX, United States

**Keywords:** human papillomavirus, community health workers, Hispanics, healthcare providers, sexual health

## Abstract

The Human Papillomavirus (HPV) is the most common sexually transmitted infection and nearly every person who is sexually active will get HPV at some point in their lifetime without having the HPV vaccine. Healthcare Providers (HCPs) and Community Health Workers (CHWs) play an essential role in promoting the HPV vaccine and providing education about HPV in communities. Three focus groups with CHWs (*n* = 17) and HCPs (*n* = 7) were conducted and led by trained facilitators. In addition to participating in the focus group, CHWs and HCPs completed a brief questionnaire. Focus groups were voice recorded and transcribed for qualitative analysis. Independent coders conducted content analysis to identify the salient themes of the focus groups. Several important findings emerged from this study highlighting the barriers to HPV knowledge, gaps in the self-perceived role of HPV cancer prevention, and opportunities to action. Financial, knowledge, patriarchy, behaviors, attitudes, and fears were identified as the perceived patient-related barriers to promoting HPV cancer prevention. Both CHWs and HCPs explained that their female patients are often discouraged by their husbands from seeking out sexual health-related healthcare. Finding suggest the need for community tailored education on HPV and “best practice” trainings for HPV prevention that is applicable to both CHWs and HCPs.

## Introduction

The Human Papillomavirus (HPV) is the most common sexually transmitted infection with as many as 43 million infections in 2018 (1). Nearly every person who is sexually active will get HPV at some point in their lifetime without the vaccine (2). HPV contains more than 150 different DNA related strains. Most HPV strains are asymptomatic and the infections will resolve on their own and will not cause health problems. However, some HPV strains be considered “low risk” and result in genital warts, while other HPV strains can be considered “high risk” and have been associated with cancer or other health problems if unnoticed. The HPV vaccine protects individuals from the high-risk strands that cause genital warts and various HPV-related cancers (1). Cervical cancer among cancer of the vulva, vagina, penis, anus, throat, tongue, and tonsils have been attributed to HPV (1). Screening and vaccination are the safest and most effective ways to be protected against HPV-related illness (3). Proper screening differs by age, gender, and previous screening results (4–6). Screenings are gender specific in detection, with women being able to get an HPV test to look for the virus and a Pap test for precancers cell changes, men do not have any approved test of HPV (7, 8). For early detection of cancer, diagnosis and treatment, the public needs to be educated on prevention efforts, health promotion, and the administration of the HPV vaccine (9). Without the education and knowledge on what HPV is and how to be protected against it, HPV infections can lead to more serious issues and go undetected until a cancer diagnosis which could lead to a poorer prognosis.

The U.S.-Mexico border region, where HPV prevalence for any subtype has been found to be 53.2%, is reported to have the highest cervical cancer rates in the United States, with 8.4 cases of cervical cancer for 100,000 females (10). This number is higher for Hispanic women as compared to their White, non-Hispanic counterparts at 6.5 per 100,000 (11). (El Paso, Texas), is one of the largest metropolitan cities on the U.S.-Mexico border, with 82% of its 839,238 inhabitants of Hispanic origin (12). Adolescents in (El Paso, Texas), have the highest rate of first-dose HPV vaccine uptake in Texas and one of the highest in the country (13). This suggests the presence of facilitators for first-dose HPV vaccine uptake in the population of predominantly Mexican origin in (El Paso, Texas) and indicates that this specific location may provide important information in the study of first-dose vaccine uptake in children and young adults. However, it is important to recognize that the COVID-19 pandemic has been identified as a significant barrier in health-seeking and obtaining non-COVID-19 related medical services (11). Data is needed to understand how COVID-19 has specifically affected HPV vaccine uptake and HPV-related education.

To further understand the barriers to HPV education delivery and HPV vaccine uptake in (El Paso, Texas), it is important to understand that sexual health education plays a key role in both. Sexual health education in Texas is focused on abstinence-only education. Health literacy is essential in lowering STI rates, including HPV (14). The (El Paso, Texas) Independent School District (EPISD) is the 12th largest district in Texas, with nearly 50,000 students. The sexual education curriculum in EPISD, which is State mandated, focuses solely on abstinence, considered by the State to be 100% effective in preventing pregnancies, STIs, and any emotional trauma (15). The curriculum used at EPISD as well as the lack of sexual health resources outside of the school setting may present barriers to increasing health literacy in the community (16). With 80.3% of the (El Paso, Texas) community reporting only having a high school degree, this abstinence-focused education provided by school districts may be the only sexual health education (El Paso, Texas) residents will encounter in their lives (12). The public health care system, through Community Health Workers (CHWs) and other healthcare providers, therefore, may be left the task of increasing health literacy by supplementing the knowledge of people in the community.

CHWs, also known as promotors/as de salud, play an essential role in educating the community, promoting healthy behaviors, and providing resources. These individuals have been referenced as paraprofessionals who have a deep understanding of the community they are serving and whose primary goal is to provide healthcare resources and education to said community (17). CHWs are trusted individuals in their respective communities and act as a bridge between the public and health providers (18). Their role is integral in removing barriers that prevent the community from seeking out resources and connecting them with the proper health care services (17, 19). CHWs promote meaningful changes in the health behaviors of the community, as they are in close contact and provide education to the communities they serve (20). The low economic and health insurance rates in communities have further increased the need for CHWs and their connection to health-related services (21). CHWs are doing essential work for health promotion and prevention in these communities with different and special needs (22, 23).

CHWs act as vital links to the health care system by providing referrals, transportation, food assistance, social support, and removing situational barriers, among other services. Texas is one of 16 states in the U.S. where CHWs are required to follow a state-wide training requirement through state government programs, colleges, or training centers that emphasize health education and community outreach (24). Programs have diverse curricula, require different durations of training, and vary in methods (24). To obtain this certificate in Texas, individuals must complete an approved 160-h training program. Their education does not stop here as they are also required to renew their certification every 2 years with continuing education credits. CHWs’ efficacy is debated in the field, but their contributions are well documented and have been seen to improve access, the education and behaviors of their communities for specific conditions (asthma, hypertension, diabetes, HIV/AIDS, cancer screening, vaccines, maternal and child health) (25). CHWs have a long history as salient links for low-income members of the border community to health services and information.

Other barriers traditionally overcomed by CHWs are language differences between providers of services and clients. The U.S. Census has a poverty threshold of $31,661, and (El Paso, Texas), has a poverty rate of 18.8%, with a per capita income average of $21,683 (12). Texas also reports having 18.4% uninsured individuals, of which 10% are Hispanic (12). (El Paso, Texas), reports having 22.1% uninsured individuals, which is estimated to have risen after the COVID-19 pandemic (26). (El Paso, Texas) is one of the 22 cities where Spanish is predominantly spoken to English by 70–80% of the population (27). The role of the CHWs in this community is not only to educate the community and provide resources, but to make sure they are linguistically and culturally appropriate for the individual (28, 29). CHWs are often found in locations where healthcare is limited, and this being a community made up of majority Hispanics and immigrants, with high poverty rates, low rates of education, and low insurance rates.

Healthcare providers (HCPs) (i.e., physicians, nurses, nurse practitioners) are other key figures in health care education who receive a different education and training than CHWs. Curricula vary as they are based on the degree being obtained. HPV education and training is inconsistent with knowledge gaps across the various healthcare professions as it is not often a focus in the various curricula (30). Both CHWs and HCPs deliver care in the community in two different and complementary ways. While CHWs take on the first contact with the individual, build rapport, gain trust, and connect individuals to services who otherwise would not receive these services, HCPs have the role of service delivery. HCPs are the next line of contact with individuals, and they have the responsibility of not only delivering the services but of providing their patients with a more extensive health education. It is this provider-patient relationship that makes patients gain more trust in the healthcare system, perceive a greater quality of care, and increase frequency of visits (31).

HPV vaccinations have been received with low enthusiasm and acceptance by different communities. The latest vaccination rates from 2020 report that only 54% of adolescents have received two or three doses at the recommended time (52% for males; 56% for women) (32). Hispanic adolescents between ages 13–15 have received two or three doses of the HPV vaccine at 58% while Non-Hispanic Whites report a 51% vaccination rate (32). Due to this low reception of the HPV vaccine, it is important for the public health sector to identify the barriers to vaccine uptake and understand the different roles CHWs and HCPs play in increasing the rates of the vaccine.

The (El Paso, Texas), a border town with an 80% Hispanic population, is a prime location for exploring the barriers to HPV vaccination uptake. HPV-data and research are prominent in the health care field yet the frequency with which they reach the community may not be sufficiently efficient to improve knowledge gaps or change attitudes and perceptions (33). Gaps in knowledge include topics about HPV strains that lead to cancer, HPV-related cancers, information on vaccine effectiveness, vaccine side effects, abnormal pap smear tests, and sexual promiscuity stigmas (34). The HCP’s role in the HPV-vaccine uptake is key in closing the gaps in knowledge of the individuals they serve. Literature indicates that HPV-vaccine uptake can have up to a foldfold increase if there is a strong provider recommendation to the parents (35).

The current study is part of a 5-year behavioral research project that will assess HPV-related knowledge, attitudes, and practices in a Hispanic community, identify barriers and facilitators of vaccine uptake, and develop and utilize interventions to improve vaccination, screening, and health literacy. The project follows three aims: (1) Identify predictors of HPV vaccine acceptability, knowledge, and screenings (VAKs) among Hispanic adults, (2) assess the practices and behaviors of emerging and current healthcare providers, followed by an intervention to improve provider recommendation and patient communication strategies, and (3) develop, implement, and deploy a culturally-tailored, bilingual, intervention to increase HPV vaccine uptake, screenings, and critical health literacy across a broad age range (26–45).

This focus group falls under aim 3 and is one of three focus groups (HPV-associated cancer survivors and caregivers; CHWs/HCPs; youth and adult males) that will be used to inform the intervention (36). These three focus groups in combination with the VAKs community sample study will come together to inform the intervention of a Randomized Clinical Trial (RCT) that will attempt to vaccinate 400 Hispanic adults during Fall 2023. The aim of this focus group is to understand perceptions about the roles of CHWs and HCPs in increasing HPV-vaccine uptake. Knowledge, attitudes, and practices related to Human Papillomavirus (HPV), the HPV vaccine, and HPV-related cancers were examined among CHWs and healthcare providers HCPs in a U.S.-Mexico border city. The goals of the current study were to: (1) assess HPV-related knowledge, attitudes, and practices, (2) identify barriers and facilitators of vaccine uptake; and (3) assess the practices and behaviors of CHWs and HCPs.

## Methods

The study was cross-sectional and qualitative in nature. Data was collected through three online focus groups of CHWs and HCPs. Focus groups are considered effective research methods, and, in their online modality, online focus groups remain effective and provide a convenient flexibility (37). During a focus groups, participants are invited to come together and increase understanding of experiences. Interactions among groups members can provide invaluable insight, ideas, and bring new questions and issues not previously explored. Therefore, focus groups are a strong methodological tool used in health promotion and especially helpful in planning, designing, and implementing interventions geared to health promotions efforts. The study utilized purposive sampling, selecting these two groups due to their shared characteristics and relevance to the end goal of the project.

CHWs and HCPs were recruited to participate by local partner agencies in (El Paso, Texas), the project manager, and CHWs working with the research team through flyers and word of mouth. These local agencies included federally qualified health center, community-based organizations, local department of public health, the Mexican consulate, local cancer care centers, and the primary institution. These agencies are committed to understanding the health needs of the community and to increasing health access, and they supported the aims of the research project. Willing participants were invited to take part in a brief survey followed by a focus group through the online service Zoom (2020). The initial survey asked participants to answer demographic questions and questions about their HPV knowledge, attitudes, and practices. Upon completion, participants were given the focus group information and invited to participate. During these Zoom sessions, participants were first introduced to the research team and to the navigation tools that would be used for the group discussions; ground agreements for the discussion were established; and a detailed explanation of the informed consent was given, after which participants submitted them electronically. Once consent forms were submitted, the recording of the session began with the participant’s permission, and facilitator began asking the focus group questions to facilitate discussion. Participants were asked about their knowledge about HPV, their knowledge about the HPV vaccine, their HPV health messaging, and recommendations to improve HPV vaccination uptake. Their participation was voluntary and anonymous and had an average duration of 1.5 h. Two researchers trained in Public Health and Social Work facilitated the groups using a structured open-ended question guide. Data were gathered between April and June 2020.

The first two focus groups were conducted in Spanish and in total included 17 CHWs (*n* = 17). Both groups were combined in data analysis due to similarity in scope and group characteristics. The third focus group was with HCPs and was conducted in English. The third focus group occurred over the span of two different dates. On the first date, seven HCPs (nurses, physicians, health professionals) (*n* = 7) were part of the focus group with the same online modality. Due to time constraint and interest for the participants to finish the entirety of the questions, a second date was added for the focus group. On the second date, only four (*n* = 4) of the original participants were able to come back and finish the study. After the focus groups the recordings were transcribed by trained transcribers from the research team, who protected the anonymity of the participants. Upon completion of the focus groups, participants were given $30 gift cards to compensate for their time and participation.

### Data analysis

A qualitative coding manual adapted from Saldaña (38) was prepared by project research assistants to facilitate and standardize the team coding process of the qualitative data. Participant responses were organized in a template created through Microsoft Excel to facilitate manual coding. Three coders from the research team independently employed a pre-coding technique to help filter important information through highlighting, underlining, or coloring salient words and/or phrases in individual participant responses (39). This process is important to facilitate the coding process to follow. Coding was done manually on an excel sheet as well as with the use of NVivo Software, a qualitative and mixed methods data analyzing software which permits for the identification of themes, visualization tools, and draw conclusions (40). Data were then interpreted through the creation of preliminary codes using affective methods (i.e., emotion and value coding) and elemental methods (i.e., descriptive and NVivo coding). An emotion code included participant’s sentiments, feelings, reactions, excitements, and sensations. Value codes included coding participant values, attitudes, and beliefs. Descriptive codes were short phrases or words that described the content. And finally, NVivo codes were coded in the software program to identify if participant’s own words seemed to be an important direct quote. Pre-coding was the first stage to data analysis, in which coders would listen to the audio-recording while reading along the transcription. Coders were instructed to retain from coding and use this stage to make notes of areas they thought were relevant and would require further attention during the coding rounds. During coding rounds, coders were instructed to code comment by comment, look for salient sections, quotes, and/or sentences that stuck our to them. All of their codes were tracked through Microsoft Excel. After a pre-coding round and two independent coding rounds, coders would share their codes and work simultaneously to identify differences in codes. This process, known as triangulation, was used to reduce bias and enhance intercoder reliability and reduce error in the coding process. During this process, the coders would work together to analyze the final codes for patterns, identify which connections and create categories. Final codes and categories were presented to the project research team for theme extraction. Disagreements were discussed and resolved during coding meetings. Qualitative analysis focused on four domains: (1) general HPV knowledge; (2) access to healthcare services related to HPV prevention; and (3) HPV health promotion efforts.

## Results

For participant demographics (see Table 1).

**Table 1:** Participant demographics.

Community health workers (*n* = 17)		
Gender, n (%)	Female	14 (82)
	Male	3 (18)
Ethnicity, n (%)	Hispanic/Latino/Mexican-Origin	15 (86)
	Non-Hispanic/Latino	2 (14)
Primary language spoken at home, n (%)	Spanish	10 (59)
	English	2 (12)
	Both	5 (29)
Time employed as CHW, n (%)	Less than 1 year	6 (35)
	1–4 years	8 (47)
	5–9 years	2 (12)
	Prefer not to answer	1 (6)
Population served, n (%)	Children (<18)	3 (18)
	Young adults (18–24)	4 (25)
	Adult ages (25–64)	6 (36)
	Older adults (65+)	4 (21)
Health care professionals (*n* = 7)		
Gender, n (%)	Female	6 (86)
	Male	1 (14)
Ethnicity, n (%)	Hispanic/Latino/Mexican Decent	7 (100)
	Non-Hispanic/Latino	0 (0)
Primary language spoken at home, n (%)	Spanish	1 (14)
	English	5 (72)
	Both	1 (14)
Time employed as health professional, n (%)	1–4 years	1 (14)
	5–9 years	1 (14)
	10+ years	5 (72)
Current role, n (%)	Nurse	5 (72)
	Medical doctor/Physician	1 (14)
	Health professional	5 (14)

Reporting focuses on these CHWs and HCPs regarding the salient similarities and differences in their roles, knowledge, and challenges related to promoting HPV cancer prevention in the Hispanic U.S-Mexico border community of (El Paso, Texas),

The roles CHWs and HCPs saw for themselves in HPV cancer prevention shared similarities, however, were distinct in some of their approaches (see Table 2). Additionally, both CHWs and HCPs identified the need for HCPs to be more proactive and intentional in their role in HPV health promotion. HCPs are viewed as authority figures by the community and could play an active role in providing information to patients. HCPs recognized that they need to take an active role, and mentioned community partnerships and educational campaigns as a good avenue for this. Regarding the HPV vaccine, all CHWs (*n* = 17) and HCPs (*n* = 7) stated that they would recommend that patients receive it. Two HCPs reported:

**Table 2:** Self-perceived roles in HPV cancer prevention.

Community health workers	Healthcare providers
Educate—first self, then community	Educate—first self, then healthcare workers, then community
Education strategy: (a) debunk myths, (b) clarify misinformation, (c) “spread fear” about consequences of HPV (to motivate parents to vaccinate their children)	Education strategy: (a) build rapport with patients, (b) create friendly, caring clinical environment (to encourage patients to ask questions and be open to communication about HPV and sexual health)
No mention of recommending HPV vaccine	Make “strong recommendations” to get the HPV vaccine

“If we make strong recommendations on the provider side, if the provider and the staff is knowledgeable, it’s up to date with the most current information, then we are making those really strong recommendations and not be so lackadaisical: ‘well there is a vaccine if you want it, if not…’ you know… because… they come to us, they come to us because we are the professionals. We were supposed to know this stuff.”

“Having advanced practitioners or nurse practitioners go out to the community and educate patients and geared towards schools, maybe some sort of school age children, High Schools, elderly, adults, parents, let’s have something for the parents, education for the parents, go into the community.”

CHWs displayed a range of awareness and knowledge about HPV, some evidencing strong knowledge of HPV-associated risks, such as cervical, uterine, and oral cancers, and others having only basic or very little knowledge of HPV stating “It is one of the most common STI’s. I do not really know too much about it besides that.”

CHWs gained their knowledge of HPV in different ways, the majority learning of it through HPV-affected family, friends, or clients; some gaining awareness through school or work-related trainings or courses; and one learning about HPV through a gynecologist. During the initial pre-focus groups survey, when asked “Do you believe you are properly informed about HPV?” one CHW strongly agreed, five agreed, nine were not certain, and two did not agree.

HCPs displayed clinical knowledge of HPV, stressing its association with cancer and genital warts. They reported a variety of ways of having learned about HPV, including experience in school and clients with HPV infections, pediatricians or pharmaceutical representatives, or self-education. “I first learned of HPV when the pharmaceutical company started coming around and talking about it, but further than that, I went ahead, and I educated myself with the content.” When asked, “Do you feel adequately informed about HPV?” during the initial pre-focus group survey, one HCP strongly agreed, three agreed, three were undecided.

CHWs and the HCPs identified many similar perceived obstacles in their efforts to prevent HPV cancer in the Hispanic community of (El Paso, Texas). These obstacles related to the community members’/patients’ financial situations, knowledge of HPV, influence of patriarchy, and attitudes. Additional patient-related barriers mentioned by HCPs were behavior and fear (see Table 3).

**Table 3:** Patient-related barriers to promoting HPV cancer prevention according to CHWs and HCPs.

	Community health workers	Healthcare providers
Financial	Lack of health insurance, (b) lack of resources to pay for services. “Basically where [vaccines] are free… only certain ages are included like 9–21 years if you are older than 21 they do not have it for free. This is when a lot of people do not get it because they do not have money to pay for it.”	Lack of health insurance, (b) lack of resources to pay for services. “If you do not have insurance the price is really high.”
Knowledge	Lack of information about HPV and HPV vaccine, (b) misconceptions about HPV and the HPV vaccine, (c) lack of information on community resources that offer sexual health services. “I have even had some talks with parents of patients in the health department and they said not to give their children the vaccine because for them it was inviting their children to start a sexual life.” “*Platicando con una señora… ella me decia ‘pero la vacuna solamente es para mujeres.’ Ella pensaba que era solamente para mujeres, no para los hombres tambien*.” (“Talking with a lady… she told me ‘but the vaccine is only for women.’ She thought it was only for women, not for men as well.”)	Lack of information about HPV and HPV vaccine, (b) misconceptions about HPV and the HPV vaccine. “…it’s not treated as a vaccine like we do now with measles and chickenpox or anything like that. It’s more like a morality, it’s more of my child you know… is now gonna be sexually active”
Patriarchy	*Machismo* (seen in male partner refusing to let female partner go to doctor because her intimate parts would be seen by doctor). “I have heard that they do not get the pap-smear because they are embarrassed or their husband does not let them.”	Strong adherence to gender roles (women submitting to male partners who do not want them to see doctor and having to go in secret for sexual health-related care).
Behaviors	No mention of behaviors.	(a) Practice of not openly discussing women’s sexual health, (b) Practice of not seeking out healthcare unless already sick.
Attitude	Stigma (associating HPV as a condition that only occurs in “promiscuous” women). “I have heard that this only happens to people who have a lot of sexual partners, specifically women.”	Stigma (associating HPV prevention with sexual activity—especially in children/youth—and sexually transmitted infection).
Fear	No mention of fear.	Fear about side effects (of vaccine) that they have heard about through non-reliable sources. “…they are skeptical, they are afraid to go out …they are not at school, it’s not a required vaccine you know”

Non-patient-related obstacles mentioned by HCPs were high cost of HPV vaccines, vaccine accessibility, and inconsistency in practices and information provided by HCPs, which causes distrust in patients and results in patients not seeking out medical care, including HPV-related care. One HCP mentioned Providers giving unnecessary pap smears as an example of this last obstacle.

“Primary care providers do not carry the vaccine, so now they have to out to either a pharmacy or try to seek it somewhere else which to me, if you don’t have it there, you don’t capture them when they are at the visit. How likely are they to follow up, or how soon are they likely to seek out this vaccine and get it done?”

## Discussion

The current study provides unique insight into the barriers that CHWs and HCPs perceive are faced by Hispanic communities they serve. These include financial challenges, knowledge about HPV, and socio-cultural factors such as patriarchy (e.g., Machismo). Several important findings emerged from this study which highlight the need for community-tailored interventions. For example, CHWs and HCPs both expressed financial concerns related to patients lacking health insurance and other resources to obtain services. These findings were expected given that (El Paso, Texas), has a poverty rate of 18.8% (12), and recent estimates suggest that 22.1% of (El Paso, Texas residents), are uninsured (25). These findings also reflect similar findings from literature which report financial barriers as the most significant barriers to care in underserved populations (39). Identifying resources to counter financial barriers to HPV vaccination and to improve access to HPV education and healthcare is warranted.

Another important finding that emerged was that both CHWs and HCPs discussed knowledge barriers related to countering misinformation and misperceptions about HPV and the HPV vaccine. These findings are congruent with a review by Zimmet et al. (41) reporting that most fears associated with the HPV vaccine are associated with false information or fabrications and suggests that interventions should focus on educating HCPs to improve their approaches toward communicating the safety and benefits of HPV vaccination to parents and patients, as well as the risks of non-vaccination. Communicating the benefits and risks associated with vaccinations is challenging and must be approached with caution. For example, paradoxical effects were reported by Betsch and Sachse (42) when investigating strategies to debunk vaccine untruths. That is, the authors found that messages that strongly deny the risks associated with vaccination were associated with increased perceived vaccine risks (e.g., vaccine-adverse events). This finding highlights the critical need for testing messaging techniques prior to deployment to ensure that boomerang effects do not emerge.

Culture emerged as an important fact that contributes to sexual health-related healthcare. The cultural barriers of women’s sexual health not being openly discussed within Hispanic communities fosters feelings of shame and embarrassment. Furthermore, there is a notion of strong adherence to gender roles in the Hispanic community. Hispanic males are often associated with the term “machismo” which can be defined as an over masculine character that is often reported as “possessive of women” and women playing a submissive role in their relationship. Notably, our findings revealed that both CHWs and HCPs explained that their female patients are often discouraged by their husbands from seeking out sexual health-related healthcare, “The wives that could not go get the Pap smears because they were afraid of their spouse finding out.” This left women needing to keep Pap smear appointments in secret to avoid conflict with their partner.

Similarly, Guerra-Rodríguez et al. (43) investigated factors influencing HPV management in young Mexican women and reported that both men and women have erroneous knowledge about the transmission of HPV and suffer the negative psychosocial effects of the stigma of HPV including guilt and shame. Moreover, the authors reported that prescribed gender roles occur in both men and women and may reduce the likelihood of disclosing HPV infection status for fears of being seen as promiscuous.

### Limitations

This study explored small sample sizes, thus findings are not generalizable. Bias may have been introduced due to the use of self-selection of focus group members. Responses may have been impacted by the historical underpinnings of the study—it was being done during COVID-19 and via technology due to COVID-19—in that the pandemic milieu may have prevented respondents from being able to focus on the intent of certain questions, and the technology-mediated nature of the groups may have been distracting and intimidating for some. Although current literature supports the use of Zoom based focus groups. In addition, one focus group was not able to finish in one session, necessitating the reconvening of the group to finish, which resulted in the loss of three members of the group due to scheduling conflicts.

## Conclusion

The themes of this study highlight the barriers that CHWs and HCPs perceive are experienced by the community members they serve when these people are trying to access health and social services. The overarching finding points to a lack of knowledge and education surrounding HPV and preventative measures among Hispanic communities. This lack of knowledge is many times replaced by erroneous information and myths about HPV, both of which are exacerbated by stigma and cultural factors that further prevent individuals from adopting preventive practices. Another important barrier is the lack of access to health care. There is limited knowledge about the community resources available due to a lack of health insurance and finances. A high rate of uninsurance is another important obstacle that was identified. Lower rates of insurance come with medical outcomes such as being more likely to die from health-related problems, unwillingness to seek treatment regardless of the severity of symptoms, and increased emergency room services as an only option.

Identifying missed opportunities can make the difference on quality of health care services. Utilizing routine health care visits to educate about HPV, sexual health, and the vaccine to parents, youth, and children can further close the gaps of HPV vaccine uptake. However, these opportunities are being missed due to self-reported lack of knowledge from CHWs and HCPs, as well as not being fully comfortable initiating these conversations. Education is important both for the health professionals and the community, and it must be culturally tailored to meet the needs of the communities.

The perceived barriers show a need for tailored messaging and interventions for different populations. This study evidenced the need for both CHWs and HCPs to be more literate in HPV health. Although all CHWs and HCPs stated they would recommend HPV vaccination for increasing vaccine uptake, they will need to be thoroughly versed in HPV health, including an effective, community-tailored method for responding to misconceptions and myths about HPV and the vaccine. Without an increase in knowledge and available resources, CHWs and HCPs will miss opportunities to make strong recommendations for the HPV vaccine and narrow the gaps in vaccine uptake.

Additionally, HCPs everywhere need to maintain up-to-date practices and messaging regarding sexual healthcare--including preventive care and treatment--to give the best care and to engender trust in their patients, who have experienced distrust and lack of confidence in HCPs due to inconsistent messaging and practices. The Health Care System needs to address the need to develop a complimentary approach to educating for CHWs and HCPs to strengthen their ability to challenge barriers like gender roles and improves their capacity to improve community HPV health literacy that respects and draws from strengths in their community and challenges misinformation that impedes health literacy.

The health messaging and recommendations given By CHWs and HCPs Are important To consider while developing educational programs and interventions. These educational programs and interventions should Use different methods To target different Age groups, including But Not limited To social media, media outlets, and health fairs. Talking about HPV and sexual health In a Hispanic community needs To Be normalized To invite members of The community To take preventive practices and remove The myths that surround The virus. CHWs and HCPs need To continue To spread information and resources As well As encourage individuals To seek health care despite these interruptions. Closing The HPV vaccine uptake gaps will require The health care system To work together with The community.

### Next steps

As part of the 5-year behavioral research project, this focus group will inform the intervention of a RCT that will be launched during Fall 2023 to attempt to vaccinate 400 Hispanic adults. Participants will go from being unvaccinated or having an incomplete vaccination series, to be fully vaccinated during a 9-month time period where they will be followed-up by the researchers to understand how their knowledge, attitudes, and practices change over the time of the study.

With Hispanic adults being the audience, the RCT intervention will be culturally tailored. The perspectives of CHWs and HCPs will be essential in the development of these interventions, particularly as they are familiar with this population’s strengths and weaknesses.

Research should continue to be done beyond this study and continue to enrich the knowledge we have on diverse communities and their specific needs. In order to create change in health prevention and services, the different barriers that are preventing access need to be understood from a close or direct perspective. CHWs and HCPs are at the forefront of advocating and educating the community about preventive health measures and consequences associated with HPV-related illnesses. To do a better job at closing gaps and creating positive change, health care provider education needs to become a priority across settings and disciplines.

## Data availability statement

The raw data supporting the conclusions of this article will be made available by the authors, without undue reservation.

## Ethics statement

The studies involving humans were approved by the University of Texas at El Paso Institutional Review Board. The studies were conducted in accordance with the local legislation and institutional requirements. The participants provided their written informed consent to participate in this study.

## Author contributions

EM designed the study and obtained IRB approval, was instrumental in the development and revision of the manuscript. AJ analyzed the qualitative data and reported on the findings. AG helped with the qualitative data analysis and drafted the introduction and methods of the study. GF drafted the discussion and revised the manuscript. All authors read and approved the final manuscript.

## Funding

This work was supported by the National Institutes on Minority Health and Health Disparities (NIMHD) (grant no. U54MD007592), a component of the National Institutes of Health (NIH).

## Conflict of interest

The authors declare that the research was conducted in the absence of any commercial or financial relationships that could be construed as a potential conflict of interest.

## Publisher’s note

All claims expressed in this article are solely those of the authors and do not necessarily represent those of their affiliated organizations, or those of the publisher, the editors and the reviewers. Any product that may be evaluated in this article, or claim that may be made by its manufacturer, is not guaranteed or endorsed by the publisher.
